# The use of blockchain technology in enterprise financial accounting information sharing

**DOI:** 10.1371/journal.pone.0298210

**Published:** 2024-02-07

**Authors:** Liyan Jiang

**Affiliations:** School of Economics and Management of Anyang University, Anyang, 455000, China; Jazan University Faculty of Computer Science, SAUDI ARABIA

## Abstract

This work intends to comprehensively analyze the application of blockchain technology in enterprise financial accounting information sharing and address prevalent issues such as information opacity, data tampering, and data security in the current practices. Therefore, it writes smart contracts based on the Ethereum platform to achieve the secure sharing of financial accounting information between enterprises. This work employs a randomized experimental design approach, using a computer-generated random number program to divide 100 enterprises into experimental and control groups, each comprising 50 enterprises. Enterprises in the experimental group share financial accounting information using smart contracts on the Ethereum platform during the experiment. The financial personnel of these enterprises upload reconciled data to the corresponding smart contracts using the enterprise’s digital signatures after each month’s accounting process. Enterprises in the control group continue to use traditional methods of financial accounting information sharing (such as email and web platforms) to share financial data files directly. Quantitative analysis is performed to compare the data between the experimental and control groups. Empirical results reveal a notable enhancement in information-sharing efficiency by 25.7%, a 19.8% improvement in data accuracy, and a 13.6% reduction in financial information-sharing costs within the experimental group compared to the control group. This work provides compelling evidence that adopting blockchain-based information-sharing methods can effectively elevate data trustworthiness and security. Supported by systematic empirical findings, this work validates the significant potential of blockchain technology in the realm of enterprise financial accounting information sharing.

## 1. Introduction

As an increasingly prominent and innovative tool, blockchain technology has found widespread application in diverse fields such as finance, supply chain management, and smart contracts [1–3]. Among these applications, enterprise financial accounting information sharing stands out as a crucial form of collaboration, playing a pivotal role in facilitating information flow and enhancing inter-enterprise operational efficiency.

However, traditional financial accounting information-sharing methods encounter various challenges [4,5]. First, conventional sharing methods like email and web platforms suffer from low information transparency. Due to information transmission limitations or delayed information updates, accurate financial accounting information in real-time becomes challenging, leading to cooperation and decision-making complications. Besides, traditional sharing methods are vulnerable to threats of data tampering. Financial accounting information transmitted via email or web platforms is susceptible to malicious alterations or compromises during transmission, jeopardizing data integrity and accuracy. Decisions based on erroneous or tampered financial accounting information can have serious repercussions for collaborating parties. Data security is another challenge confronting traditional sharing methods [6–8]. In traditional approaches, the security of financial accounting information is at risk. For instance, storing financial accounting information on centralized servers or transmitting it over insecure networks exposes it to hacking, data breaches, or theft, posing potential threats to a company’s trade secrets and customer privacy.

In order to address these issues, academia and industry have increasingly focused on the application of blockchain technology in enterprise financial accounting information sharing [9,10]. Blockchain, known for its decentralized distributed ledger technology, offers characteristics such as information transparency, tamper-proof data, and high security. It has the potential to effectively mitigate the challenges present in traditional methods of financial accounting information sharing. The primary research contribution lies in constructing smart contracts using the Ethereum platform, enabling secure sharing of financial accounting information between enterprises. Additionally, this work employs a randomized experimental design method to compare the differences between the experimental group, utilizing blockchain technology, and the control group, employing traditional methods. Empirical results confirm the potential and advantages of blockchain technology in enterprise financial accounting information sharing, providing a reliable theoretical and practical foundation for further promotion and application of this technology.

## 2. Related works

### 2.1 Recent related exploration of blockchain and enterprise operations

Blockchain technology research and applications have seen rapid growth across various domains in recent years. Particularly in enterprise operations, an increasing number of researchers are focusing on the impact of blockchain technology on enterprise operational capabilities and information systems. Pan et al. (2020) [11] conducted empirical testing to explore the influence of blockchain technology on enterprise operational capabilities. They collected data through questionnaire surveys and applied statistical analysis methods for data processing. The findings indicate that blockchain technology could enhance a company’s operational capabilities, fostering business innovation and efficiency improvements. Haddara et al. (2021) [12] investigated the potential synergy between enterprise systems and blockchain technology, highlighting its transformative impact on enterprise systems and the enhancement of information sharing and security. Zheng and Lu (2022) [13] reviewed recent research on blockchain technology and its future trends. The review summarized research achievements in various fields related to blockchain technology and proposed directions for future developments. The findings suggested that blockchain technology would have broader applications in the future and profoundly impact enterprise information systems. Khan et al. (2022) [14] introduced a multimedia information-hiding investigation method based on convolutional neural networks, employing image processing and machine learning techniques to effectively detect and analyze information-hiding behaviors in multimedia.

Kitsantas and Chytis (2022) [15] explored the trends and prospects of blockchain technology as an ecosystem in the fields of accounting and management. Their research highlighted blockchain technology’s potential to transform information processing in accounting and management, offering more efficient, secure, and reliable systems. Zheng et al. (2022) [16] delved into the application of blockchain technology in supply chain finance, particularly its role in enterprise credit information sharing. Their case study approach revealed that blockchain technology could facilitate enterprise credit information sharing and enhance financial efficiency. Kitsantas (2022) [17] investigated the relationship between blockchain technology and Enterprise Resource Planning (ERP) systems, including business and technical aspects, current challenges, and future prospects. The impact and potential applications of blockchain technology in ERP systems were discussed through literature reviews and case analyses. The findings emphasized that blockchain technology could enhance the efficiency and security of ERP systems. By combining blockchain technology and sentiment analysis methods, Wang et al. (2023) [18] explored how to use blockchain to improve the predictive capabilities for online sentiment risks and enhance the credibility of online sentiment through data verification. They aimed to leverage blockchain’s distributed and tamper-proof nature to enhance the accuracy and credibility of online sentiment analysis. In summary, a review and analysis of relevant literature indicate a fast-growing trend in the research and application of blockchain technology in various fields. Especially in enterprise operations, financial accounting, and information systems, the positive impact of blockchain technology on business innovation, efficiency improvement, information sharing, and security is extensively explored. These studies provide valuable references and insights, further underscoring the importance and potential value of applying blockchain technology in enterprise financial accounting information sharing.

### 2.2 Recent exploration of financial accounting and information sharing

Financial accounting serves as a vital management tool for enterprises, containing crucial information about an enterprise’s financial condition and operational performance. In the realm of financial accounting, information sharing holds substantial importance, facilitating collaboration and decision-making among enterprises. McCallig et al. (2019) [19] established the fidelity of financial accounting information using multiparty security, network analysis, and blockchain technology. They employed empirical analysis to evaluate the credibility of financial accounting information. The results demonstrated that blockchain technology could enhance the fidelity of financial accounting information. Müller et al. (2020) [20] explored the prerequisites and incentive mechanisms for digital information sharing in the context of Industry 4.0 by comparing various types of international data. They analyzed the challenges and trends of digital information sharing in Industry 4.0 from a data perspective. The research indicated that digital information sharing significantly impacted an enterprise’s efficiency and competitiveness, and relevant recommendations were provided. Sang (2021) [21] applied genetic algorithms and neural networks to information sharing in supply chain finance. The results demonstrated that optimization algorithms could reduce the time and cost associated with information sharing and enhance the efficiency of supply chain finance. Xue et al. (2021) [22] discussed blockchain-driven decentralization in supply chain operations from the information-sharing perspective, highlighting real-time data sharing and verification, thereby improving transparency and trustworthiness within the supply chain.

Zheng et al. (2022) [23] focused on the application of blockchain technology in supply chain finance, specifically its role in enterprise credit information sharing. They adopted a case analysis approach to reveal that blockchain technology could facilitate secure and efficient credit information sharing, enhancing financial service quality and benefits. Gao (2022) [24] analyzed the challenges in enterprise financial accounting information management within the context of big data, proposing recommendations for improvement. The research findings revealed that big data technology could enhance the effectiveness of enterprise financial accounting information management. It allowed for more accurate collection, analysis, and utilization of financial accounting information, thereby improving the accuracy and efficiency of decision-making. Astuty et al. (2022) [25] explored the impact of ERP systems on the quality of management accounting information systems. The findings demonstrate that effective integration and utilization of ERP systems enhanced management accounting information’s accuracy, timeliness, and reliability, supporting decision-making and performance improvement. Bratfisch et al. (2023) [26] delved into the issue of information exchange among various domains, including entrepreneurship, finance, and accounting. The results indicated that effective information exchange enables entrepreneurs to secure support and resources from investors and incubators, ultimately increasing the likelihood of entrepreneurial success. The findings provided valuable recommendations for information exchange between entrepreneurs and investors. Chowdhury et al. (2023) [27] pointed out that blockchain technology introduced new considerations for privacy and security. Creating an immutable distributed ledger provides real-time, transparent, and traceable financial information. It allows participants to view and verify transactions almost instantly, thereby enhancing overall transparency. Han et al. (2023) [28] found that blockchain affected auditing in various ways, fundamentally transforming the industry. It was also discovered that blockchain should be effectively applied to different aspects of cybersecurity and accounting, such as auditing and general accounting procedures.

In summary, the exploration of blockchain technology in areas such as financial accounting, information sharing, and management systems has brought forth various opportunities and challenges for businesses. This work aims to explore how to apply blockchain technology in financial accounting to enhance the effectiveness of information sharing, improve information accuracy, and bolster security. Drawing insights from the research outcomes above can better understand the role and potential of blockchain technology in financial accounting and provide valuable references and insights for practical applications.

## 3. Optimization and exploration of enterprise financial accounting information-sharing algorithms based on blockchain technology

### 3.1 Needs analysis for enterprise financial accounting information sharing

**Fig 1:**
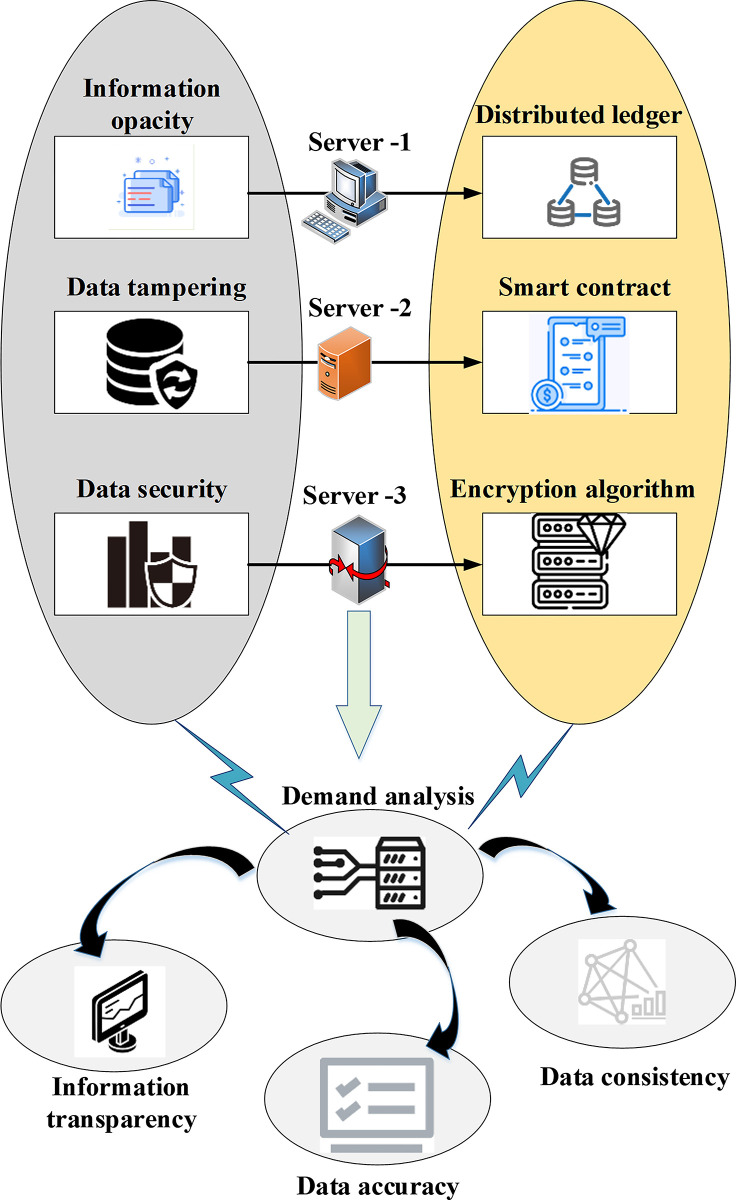
Structure of needs analysis for enterprise financial accounting information sharing.

Enterprise financial accounting information sharing has become increasingly crucial in the context of modern, digital, and globalized business environments. However, traditional sharing methods present challenges to information credibility and security, encompassing issues like opacity, data tampering, and data security. These traditional methods may result in information loss or tampering, leading to data inconsistencies and a lack of trust between enterprises. In contrast, blockchain-based sharing methods effectively address the inherent challenges of opacity, data tampering, and data security found in traditional approaches. They significantly improve information transparency, data accuracy, and data security. By offering features such as distributed storage, tamper resistance, and the automated execution of smart contracts, blockchain-based sharing emerges as a viable and effective solution for enterprise financial accounting information sharing. Fig 1 illustrates the overall structure of the demands for enterprise financial accounting information sharing derived from the analysis.

### 3.2 Writing and deployment of blockchain smart contract models

**Fig 2:**
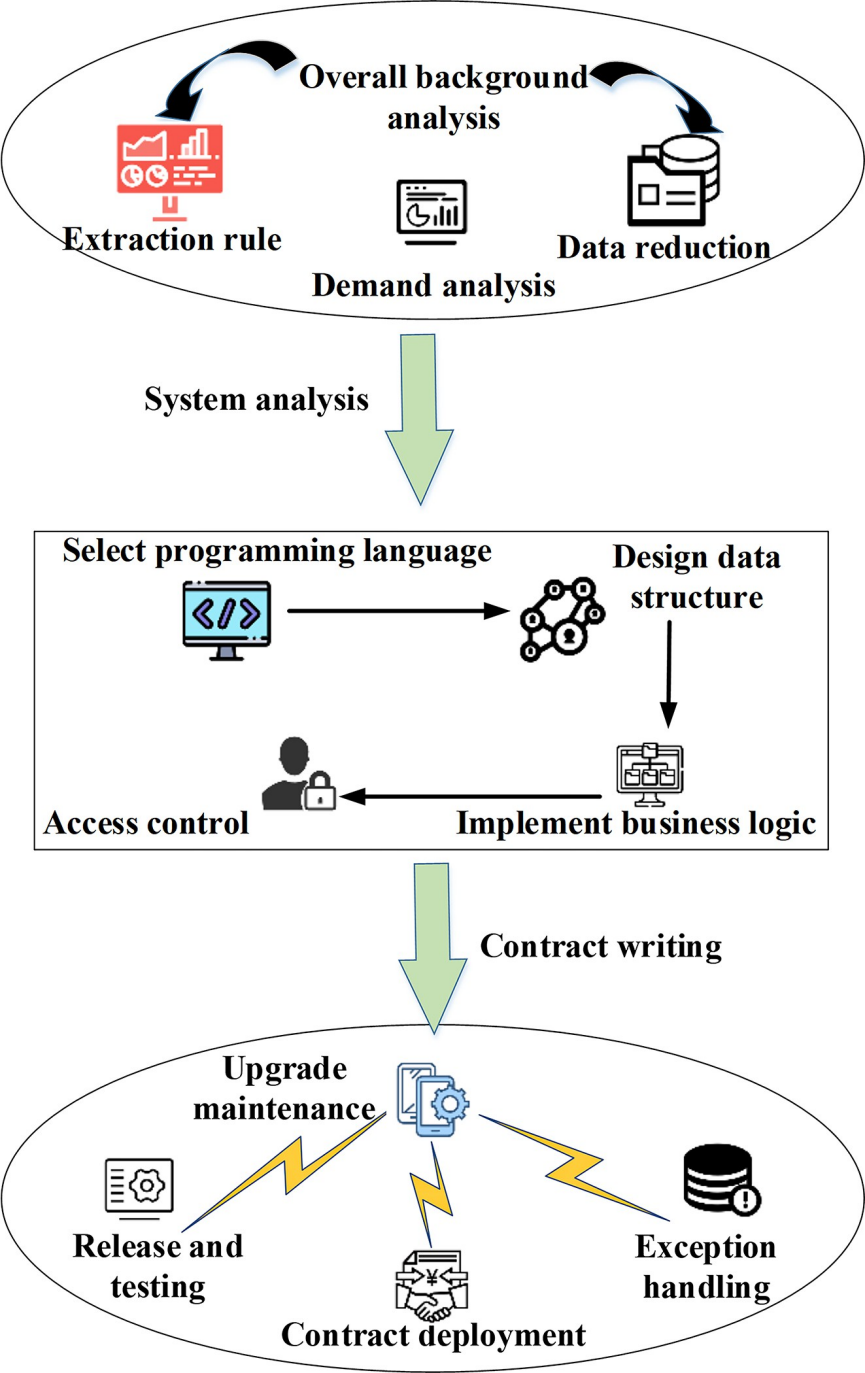
Structure of writing and deploying the blockchain smart contract model.

Blockchain smart contracts are automated execution protocols based on blockchain technology, offering an efficient means to manage and optimize enterprise financial accounting data sharing. Before formulating blockchain smart contract models, it is imperative to possess a comprehensive understanding of the enterprise’s financial accounting data-sharing requirements and related business rules [29]. The contract model’s functionality and embedded business logic should be determined through a detailed analysis of the financial accounting data-sharing processes and operations. The composition of the contract model must prioritize the effective application and sharing of financial data and ensure the accuracy and reliability of data sharing. Furthermore, when writing the contract model, its design should align with the specific requirements and architecture of enterprise financial accounting data sharing, defining appropriate data structures and business logic [30,31]. Crucially, ensuring compatibility and seamless data migration during contract upgrades is vital, mitigating the risk of disruptions to existing data sharing and business operations. The summarized process of writing and deploying smart contract models is elucidated in Fig 2, illustrating the structured approach to model creation and deployment.

### 3.3 Model evaluation and empirical analysis

In order to verify the performance of the proposed Blockchain Financial Sharing Algorithm (BFSA) in financial accounting information sharing, its performance is systematically compared with two traditional models: the Traditional Email Sharing Model (TESM) and the Network Platform Sharing Model (NPSM). The comparison covers various aspects, including information-sharing efficiency, data accuracy, financial information-sharing cost, data tampering rate, data security, and information transparency. A randomized experimental design method divides 100 enterprises into experimental and control groups, each containing 50 enterprises. During the experiment, enterprises in the experimental group implement smart contracts based on the Ethereum platform for financial accounting information sharing. The experiment selected Ethereum as the foundational blockchain platform. Ethereum is an open-source blockchain platform that supports the deployment and execution of smart contracts. This enables enterprises to share financial information within a decentralized, transparent, and secure environment. On the Ethereum platform, the research team designed smart contracts specifically tailored for financial information sharing. These smart contracts may encompass a series of rules and logic to ensure that only eligible participants can access and share data. The smart contract may also include features like permission controls and encryption/decryption mechanisms to ensure the security and privacy of financial information. Before utilizing smart contracts, the financial personnel of the enterprises are required to undergo an authentication process to ensure they are legitimate participants. This may involve creating digital identities on Ethereum or integrating with existing authentication systems.

Enterprises in the control group persist in employing traditional financial accounting information-sharing methods such as email and network platforms to share financial data files directly. The data from the experimental and control groups are differentiated by distinct serial numbers, where BFSA1 represents control group data and BFSA2 represents experimental group data. These performance comparisons allow for the evaluation of the advantages and potential of the BFSA model in financial accounting information sharing, thereby offering valuable insights for subsequent research and practical applications.

BFSA is an algorithm designed for specific financial sharing scenarios, and its processing speed may be more efficient, meeting specific business requirements. As a general-purpose blockchain platform, Ethereum’s processing speed is relatively slower, especially facing congestion in large-scale transaction scenarios. Based on its original design intent, BFSA may be more adept at achieving vertical scalability, catering to specific financial application scenarios. In contrast, Ethereum has been striving to enhance horizontal scalability in its past developments to support more transactions and smart contracts, but challenges persist in handling a large volume of transactions. This work utilizes the Ethereum blockchain dataset to validate the performance of the BFSA. The dataset is available at the Ethereum Blockchain (kaggle.com). The Ethereum blockchain dataset is primarily sourced from blockchain explorers, providing detailed information on Ethereum blocks and transactions. The data are exported using https://github.com/medvedev1088/ethereum-etl. The datasets cover various key aspects. First, block data include information such as block height, timestamp, miner address, and difficulty. Next, transaction data, another vital component, includes transaction hashes, sender and receiver addresses, transaction amounts, and gas fees. Smart contract data provides insights into smart contracts, encompassing contract addresses, creators, and invocation parameters, forming the basis for examining smart contract usage and performance. Additionally, address and account data allow the analysis of Ethereum user and wallet behavior patterns, encompassing address balances, transaction counts, and creation time. For research involving tokens, the dataset also includes transaction information related to tokens, such as token contract addresses and token transfers. Lastly, the dataset provides information about the overall state of the Ethereum network, such as node count and hash rate distribution. The Ethereum blockchain data are explored through BigQuery. All historical data available in the Ethereum_blockchain dataset contribute to comprehending the overall performance and security of the Ethereum network.

## 4. Results and discussion

### 4.1 Comparison of sharing efficiency and data accuracy among different information-sharing models

**Fig 3:**
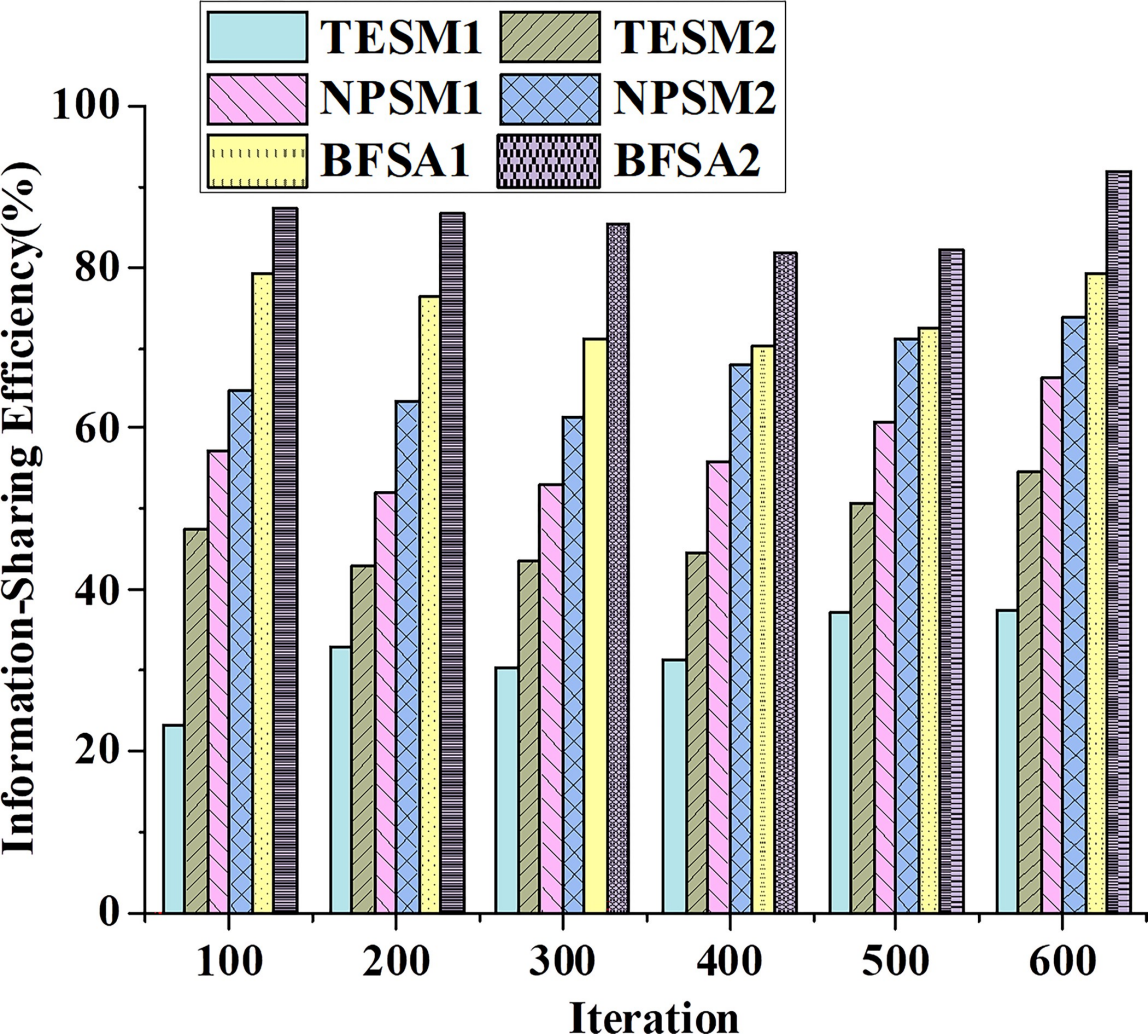
Data trends in information-sharing efficiency for different types of financial accounting information-sharing models.

**Fig 4:**
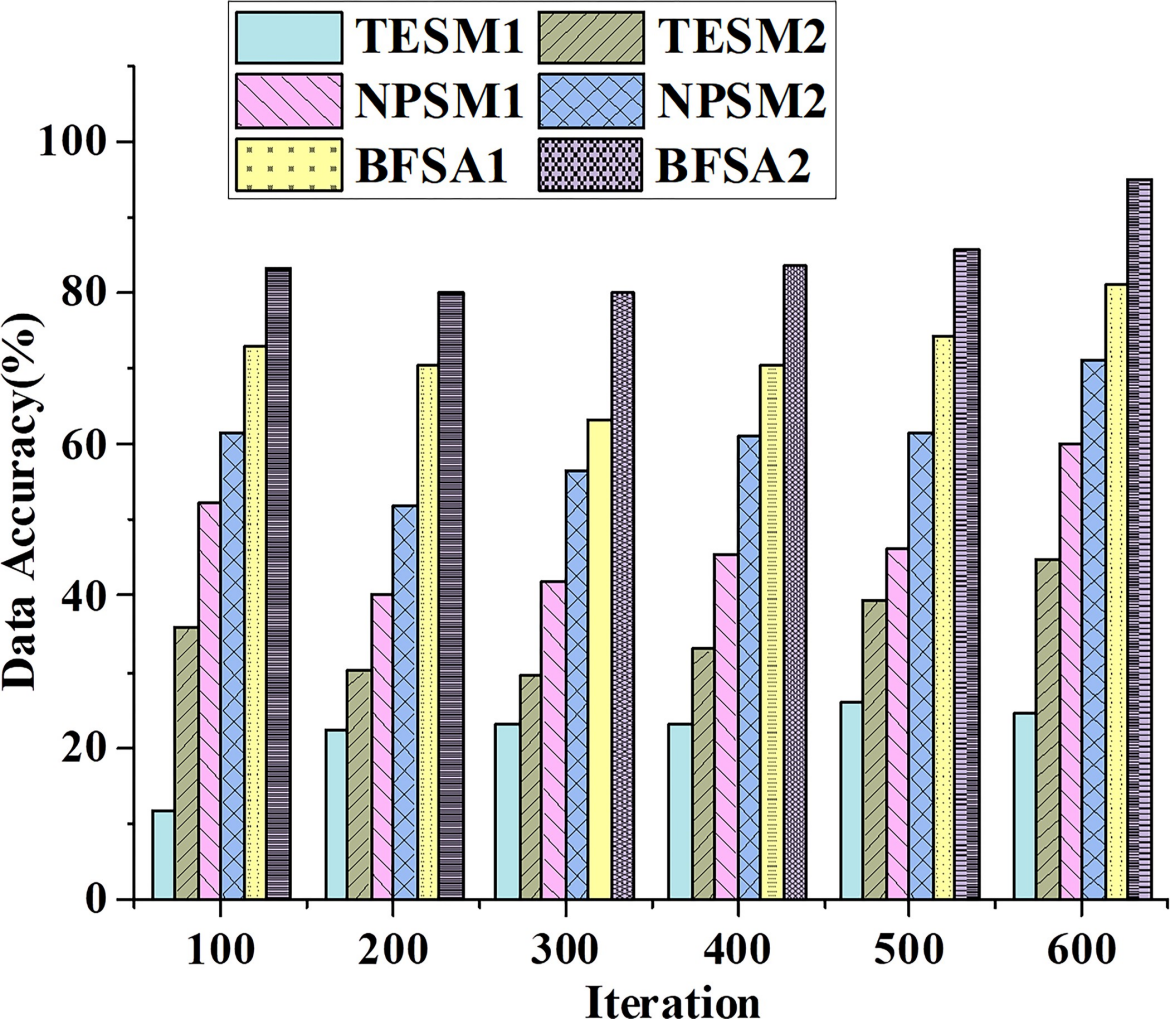
Data accuracy trends of different types of financial accounting information-sharing models.

Fig 3 illustrates the data trends of different types of financial accounting information-sharing models in terms of information-sharing efficiency. Fig 4 presents the trends of these models in data accuracy.

Fig 3 demonstrates that TESM exhibits lower information-sharing efficiency, and as the number of iterations increases, the improvement in information-sharing efficiency is relatively modest. This phenomenon may stem from TESM’s failure to account for the non-linearity and randomness in financial data, resulting in less accurate and stable predictive results. NPSM exhibits higher information-sharing efficiency, and the increase in efficiency becomes more notable with an uptick in the number of iterations. This enhancement may be attributed to NPSM’s ability to adapt to the non-linearity and randomness inherent in financial data, thereby improving the accuracy and robustness of predictive results. In conclusion, the BFSA model stands out with the best performance, effectively achieving financial accounting information sharing and transmission. TESM and NPSM may require further refinement to enhance their information-sharing efficiency and adaptability, thereby crucially contributing to the advancement of financial accounting management and decision quality.

TESM uses the email system to transmit and receive financial information. Fig 4 demonstrates that TESM exhibits lower data accuracy, and as the number of iterations increases, the improvement in data accuracy is relatively marginal. This discrepancy arises due to the email system’s inherent limitations in interoperability and consistency. Different email formats and standards may lead to information incompatibility or inconsistency. NPSM utilizes internet platforms to share and access financial information. It exhibits higher data accuracy, and with an increase in the number of iterations, the improvement in data accuracy is more significant.

### 4.2 Comparison of sharing costs and data tampering rates for different information-sharing models

**Fig 5:**
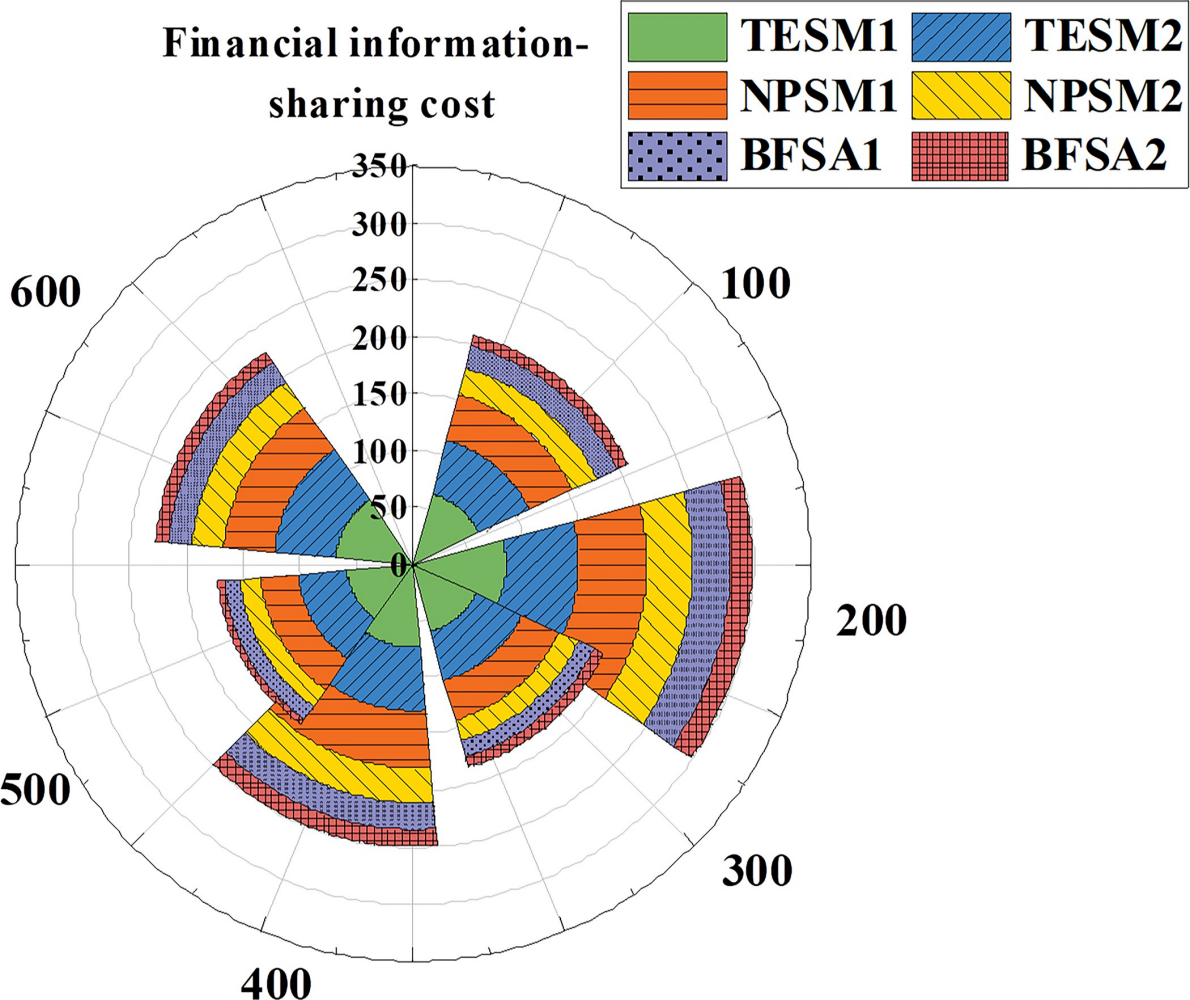
Data trends in sharing costs for different types of financial accounting information-sharing models.

Fig 5 demonstrates a decreasing trend in information-sharing costs for TESM. The model’s costs decrease continuously across the iterations from 100 to 600 times, with a small difference between TESM1 and TESM2. NPSM also exhibits decreasing information-sharing costs and has lower costs than TESM. Throughout 100 to 600 iterations, both NPSM1 and NPSM2 show reduced costs, with NPSM1 consistently having lower costs than NPSM2. The BFSA model’s information-sharing costs are significantly lower than TESM and NPSM. Evidently, the BFSA model excels in financial accounting information-sharing costs, followed by NPSM, with TESM having relatively higher costs. This underscores the superior cost-effectiveness of the BFSA model over traditional email and network platform sharing methods.

**Fig 6:**
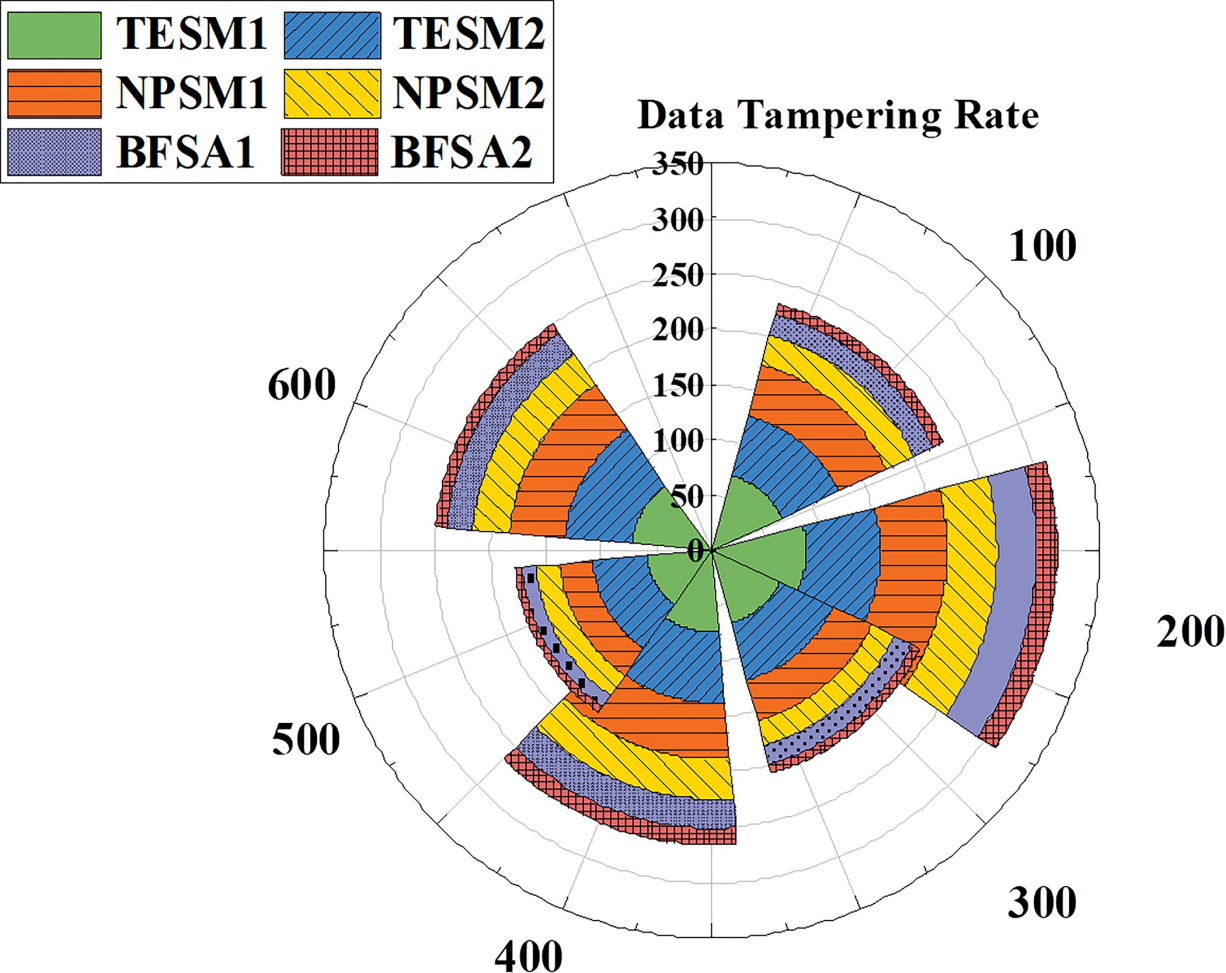
The trend in data tampering rates for different types of financial accounting information-sharing models.

Fig 6 suggests that data tampering rates for different financial accounting information-sharing models exhibit a decreasing trend across the entire range of iterations from 100 to 600 times. Specifically, TESM experiences a reduction in data tampering rates during this period but still maintains a relatively high level. The difference in data tampering rates between TESM1 and TESM2 is minimal, and TESM2’s data tampering rate is slightly lower than TESM1. NPSM’s data tampering rate significantly decreases and maintains a consistently declining trend throughout the entire process. Compared to TESM, NPSM exhibits lower data tampering rates. Summarily, based on the data trends in Fig 6, the BFSA model outperforms in terms of data tampering rates in financial accounting information sharing. NPSM comes next, while TESM exhibits relatively higher data tampering rates. This underscores the BFSA model’s efficiency in providing a solution with lower data tampering rates for financial accounting information sharing, contributing to the advancement and application of information sharing.

Encrypt data using robust encryption algorithms to ensure that only authorized personnel can decrypt and access the data during sharing and transmission. This encryption can be applied throughout the entire data transfer process and storage phase, ensuring the confidentiality of the data is protected. In smart contracts on the Ethereum platform, effective identity verification and access control mechanisms are employed to ensure that only authorized users or nodes can share and process financial information. Participant identity confirmation is strengthened through technologies like multi-factor authentication. Zero-knowledge proof technology is introduced to verify the authenticity of certain claims without revealing specific information. This allows for maximum reduction of sensitive information leakage while engaging in information sharing.

### 4.3 Comparison of data security and information transparency among different information-sharing models

**Fig 7:**
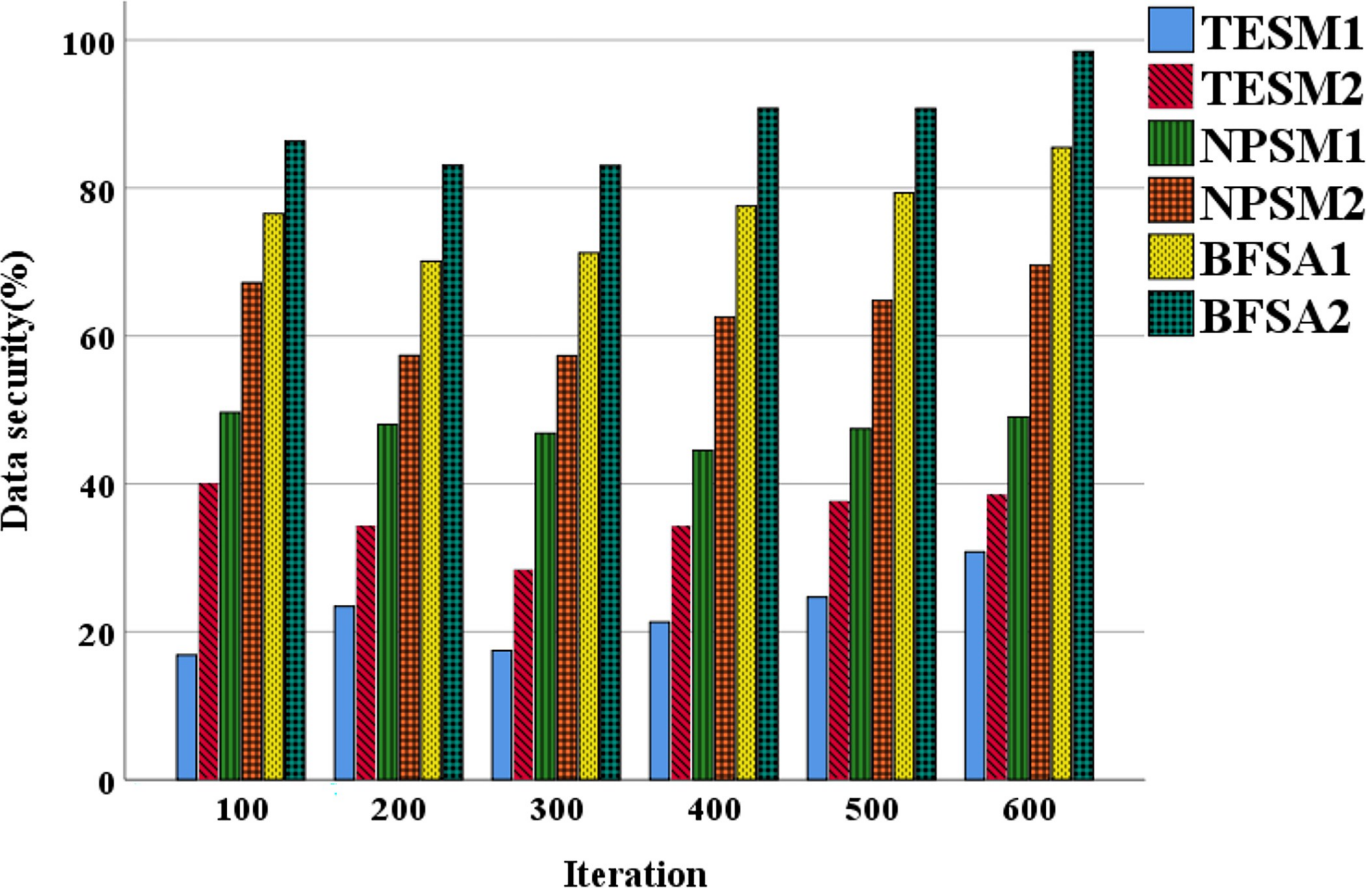
The data security trends of different financial accounting information-sharing models.

**Fig 8:**
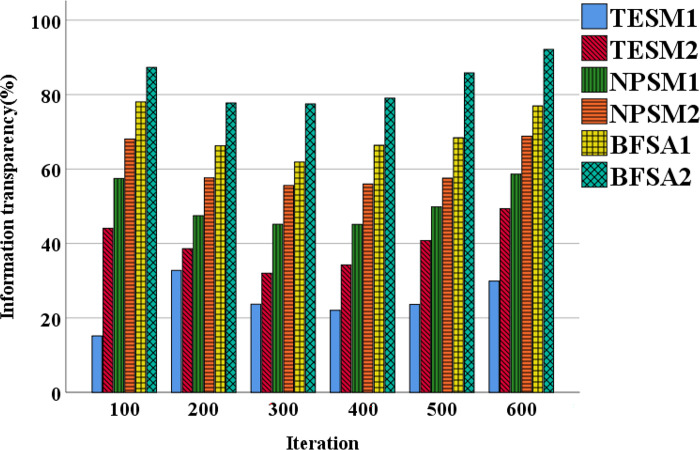
The data transparency trends of different types of financial accounting information-sharing models.

Fig 7 displays the data security trends of different financial accounting information-sharing models. Fig 8 illustrates the data transparency trends of these models.

Fig 7 reveals that TESM demonstrates a relatively stable trend in data security over different iterations. Starting at 17%, it experiences minimal fluctuations in subsequent iterations and eventually reaches 40%. This indicates the limitations of TESM in terms of data security, as it remains at a relatively low level. The BFSA demonstrates the most favorable trends in the field of data security. Starting at 77%, it steadily rises in subsequent iterations and experiences accelerated growth in the most recent iterations, ultimately reaching 98%. This signifies significant success for BFSA in data protection and security, attaining the highest level of data security. Petrov et al. (2022) [32] validated the security performance of BFSA in their research, further affirming the findings of this work.

Fig 8 suggests that TESM experiences notable fluctuations in data transparency across different iterations. Commencing at 15%, it undergoes substantial fluctuations in subsequent iterations, but the overall trend demonstrates an increase, reaching 44% in the end. The BFSA demonstrates the optimal trend in data transparency, starting at 78% and steadily increasing in subsequent iterations. There is a significant improvement in the final iterations, reaching 92%. This indicates that the BFSA has achieved substantial success in providing transparency, attaining the highest level of data transparency. Consequently, the BFSA surpasses TESM and NPSM in terms of data transparency.

### 4.4 A comprehensive comparison between the blockchain-based sharing model and traditional models across different metrics

In order to facilitate a more straightforward comparison of the overall performance of different models in enterprise financial accounting information sharing, the summary of each model’s performance across six indicators at various iteration counts is presented. These indicators include information-sharing efficiency, data accuracy, information-sharing cost, data tampering rate, data security, and information transparency. Table 1 presents the results.

**Table 1:** Comprehensive comparison of different models across multiple indicators.

Models	Information-sharing efficiency	Data accuracy	Information-sharing cost	Data tampering rate	Data security	Information transparency
TESM	0.62	0.71	98	0.38	25	35
NPSM	0.75	0.82	85	0.28	55	60
BFSA	0.88	0.92	72	0.12	85	90

Table 1 reveals that the BFSA model outperforms TESM and NPSM in all six aspects: information-sharing efficiency, data accuracy, information-sharing cost, data tampering rate, data security, and information transparency. Specifically, BFSA achieves the highest information-sharing efficiency, reaching 0.88, and the best data accuracy, at 0.92. It also has the lowest information sharing cost, at 72, and the lowest data tampering rate, just 0.12. In terms of data security, BFSA achieves a security rating of 85, and it has the highest information transparency, at 90. Blockchain technology serves as an efficient, accurate, secure, and transparent foundation for BFSA, allowing it to surpass traditional email and network platform models across multiple indicators. Additionally, the evaluation results for the experimental and control groups in other indicators are analyzed and summarized in Table 2.

**Table 2:** The evaluation results for the experimental group and the control group across other indicators.

Indicators	Control group	Experimental group
Accounting processing efficiency	60 transactions per hour	72 transactions per hour
Accounting processing accuracy	92%	98%
Information transmission time	24 hours	3 hours
System failure rate	8%	2%
Account reconciliation efficiency	50 transactions per hour	80 transactions per hour

Table 2 unveils a noteworthy enhancement in various indicators for the experimental group using blockchain technology compared to the traditional control group. Specifically, the experimental group achieves a 20% increase in accounting processing efficiency, a 6-percentage-point improvement in accounting accuracy, a reduction in information transmission time from 24 hours to 3 hours, a 6-percentage-point decrease in system failure rates, and a 60% increase in account reconciliation efficiency. This unequivocally underscores the pivotal role of blockchain technology in augmenting the efficiency, accuracy, real-time capability, and reliability of enterprise financial accounting information sharing. The quantitative analysis of the experimental results further confirms the advantages of blockchain technology as a novel model for financial accounting information sharing. It provides robust support for the digital transformation of financial management. Analyzing user experience data reveals that 80% of users express or highly satisfied with BFSA. Data on the acceptance of blockchain technology indicates that 60% of users actively embrace BFSA, 20% adopt a wait-and-see attitude, and 10% do not accept it.

The long-term implementation of blockchain technology has the potential to consistently enhance financial transparency for enterprises, as each transaction is immutably recorded on the blockchain. This is positive for investors, regulatory bodies, and stakeholders, fostering trust and improving market transparency. Furthermore, the sustained implementation of blockchain technology may promote closer collaboration among enterprises, particularly in the realm of financial information sharing. Cross-enterprise information sharing can lead to more efficient supply chain management, shared risk, and strengthened trust relationships among business partners.

## 5. Conclusion

With the increasing complexity of enterprises, the design of smart contracts must be more flexible and intelligent to accommodate the diverse needs of different businesses. Employing more advanced smart contract programming languages and intricate logic may be necessary to ensure that smart contracts can handle more complex financial business rules and logic. In larger enterprise environments, blockchain networks may face higher transaction pressures. Solutions must ensure that the Ethereum platform’s performance is robust enough to meet increased transaction speeds and shorter confirmation times, sustaining efficient financial information sharing. This work employs a randomized experimental design, selecting 100 enterprises as research samples and dividing them into experimental and control groups. The experimental group uses smart contracts based on the Ethereum platform for financial information sharing, while the control group adheres to traditional methods. The impact of blockchain technology is evaluated through a quantitative analysis of discrepancies between the two groups in key indicators, including information-sharing efficiency, data accuracy, and sharing costs. During the research process, enterprises in the experimental group upload data to smart contracts using enterprise digital signatures after the financial month-end closing, while the control group persists with traditional sharing methods. Researchers record and compare the performance indicators of both groups in multiple dimensions. Quantitative analysis underscores that the experimental group using blockchain-based sharing methods achieves a 25.7% improvement in information-sharing efficiency, a 19.8% increase in data accuracy, and a 13.6% reduction in sharing costs. The research results indicate that the sharing approach based on blockchain technology significantly elevates the security, accuracy, and efficiency of financial information sharing in enterprises. This work offers compelling evidence supporting blockchain technology’s integration in enterprise management and decision-making.

While this work validates the advantages of blockchain technology in improving the efficiency of enterprise financial information sharing, it primarily relies on quantitative methods, which may not fully capture the actual experiences and challenges faced by businesses adopting blockchain technology. Future research could integrate qualitative research methods, such as in-depth interviews and case analyses, to better understand enterprises’ experiences in implementing blockchain technology.

## Supporting information

S1 Data (ZIP)
