# Supplementary figures and images for: The use of blockchain technology in enterprise financial accounting information sharing
Source: PLoS One. 2024 Feb 7;19(2):e0298210. doi: 10.1371/journal.pone.0298210 (PMC10849433; doi:10.1371/journal.pone.0298210)

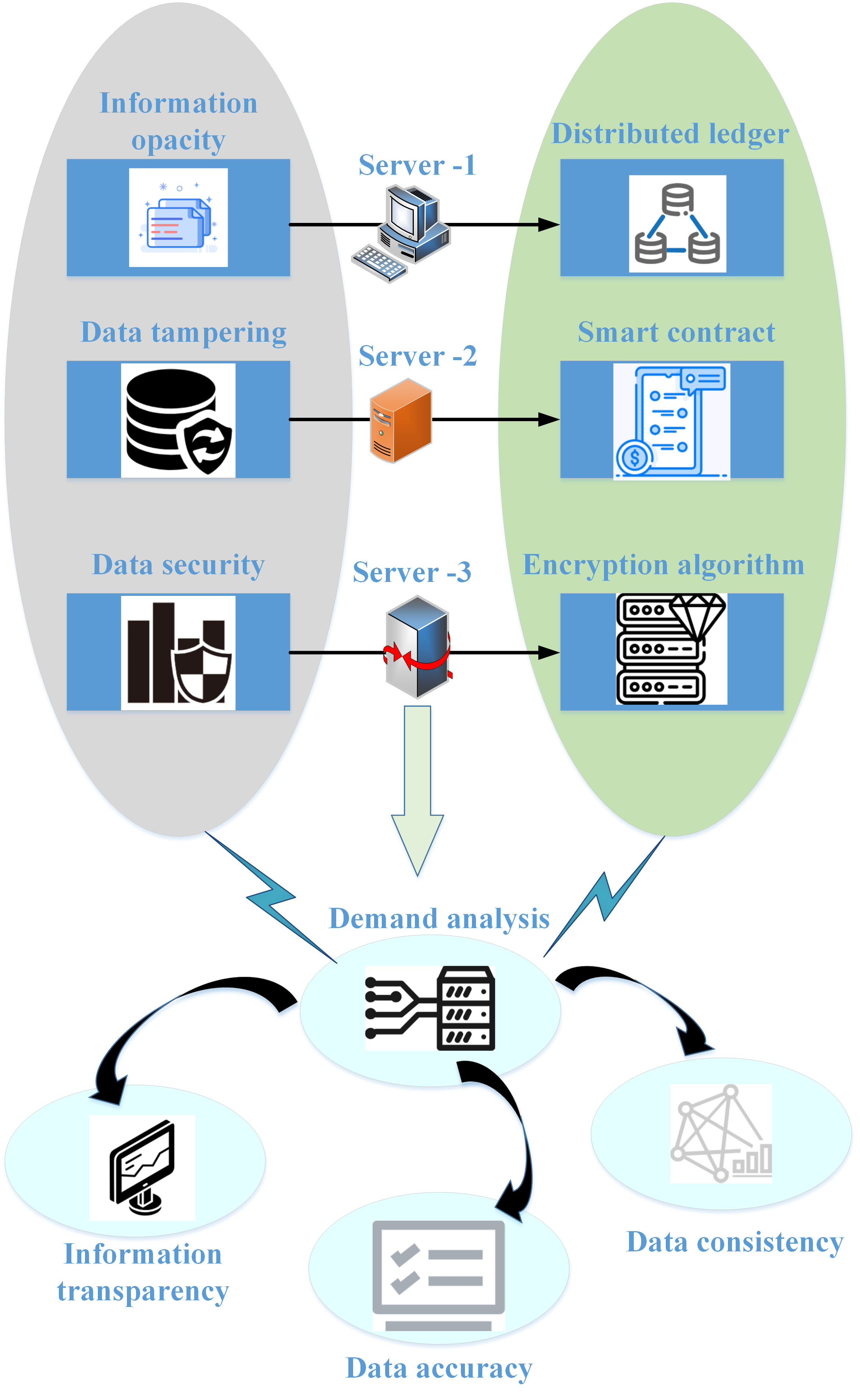

Supplement: S1 Data — (ZIP) [file pone.0298210.s001.zip › ╩2╛▌░n/Figures/figure1.tif]

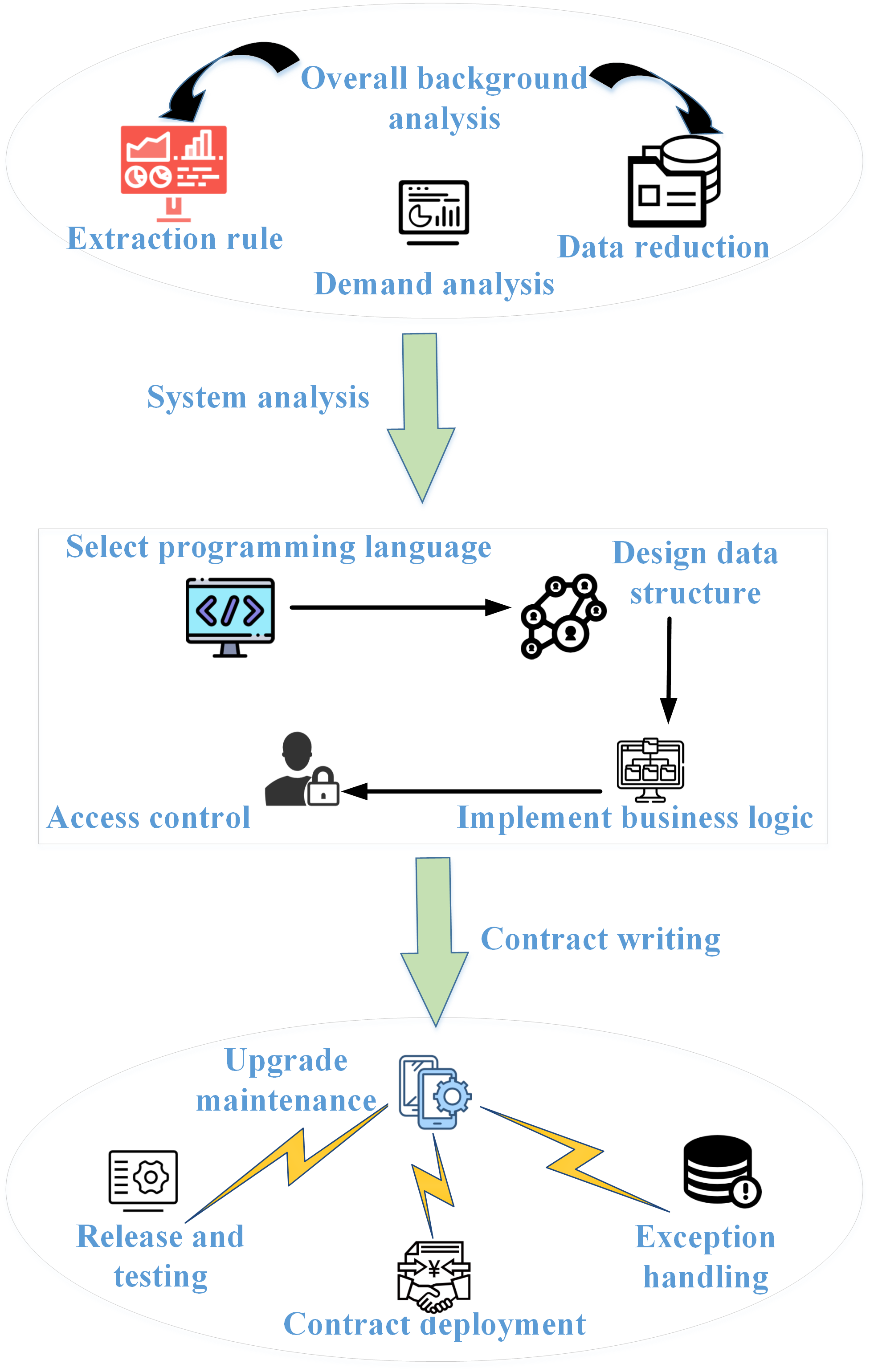

Supplement: S1 Data — (ZIP) [file pone.0298210.s001.zip › ╩2╛▌░n/Figures/figure2.tif]

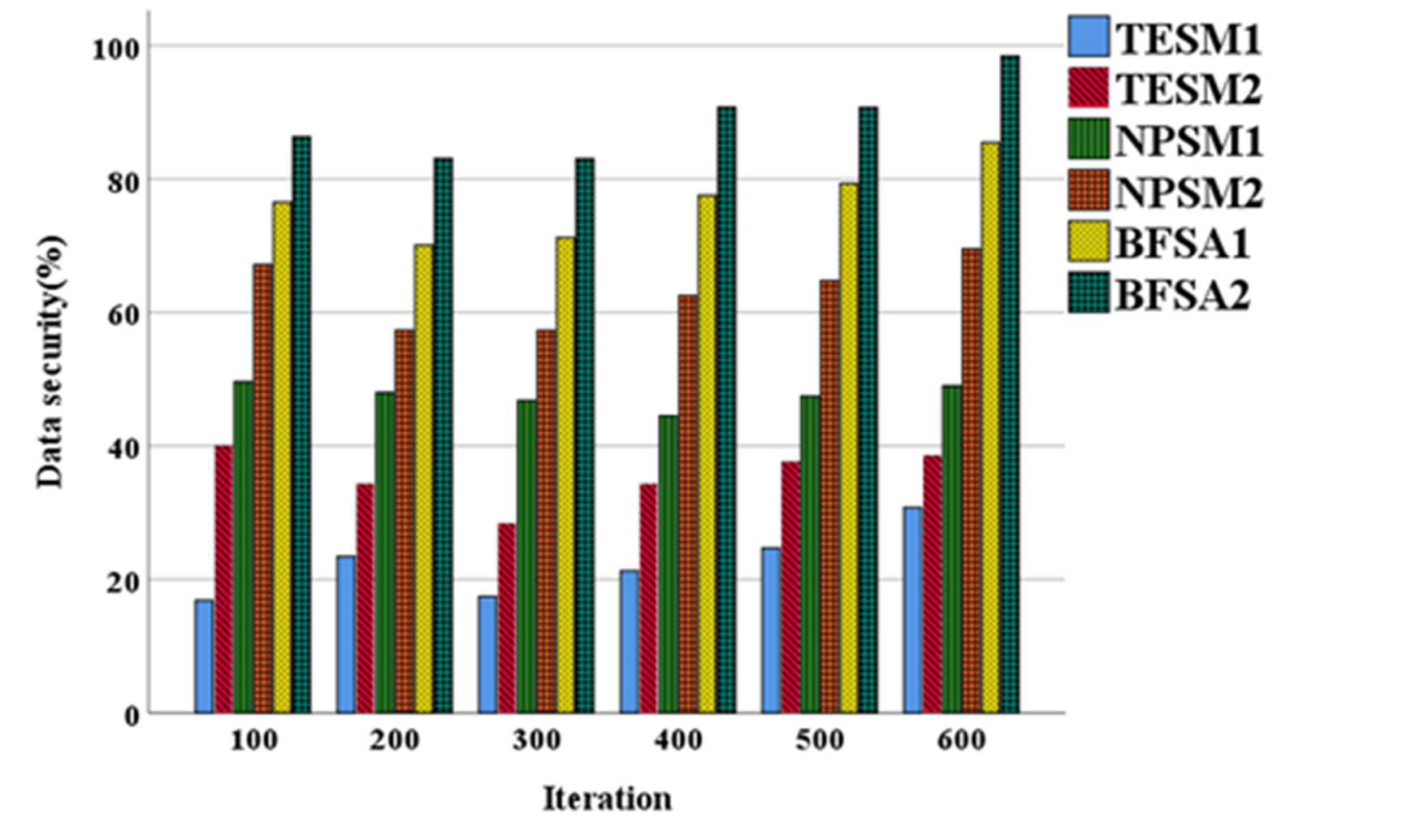

Supplement: S1 Data — (ZIP) [file pone.0298210.s001.zip › ╩2╛▌░n/Figures/figure7.tif]

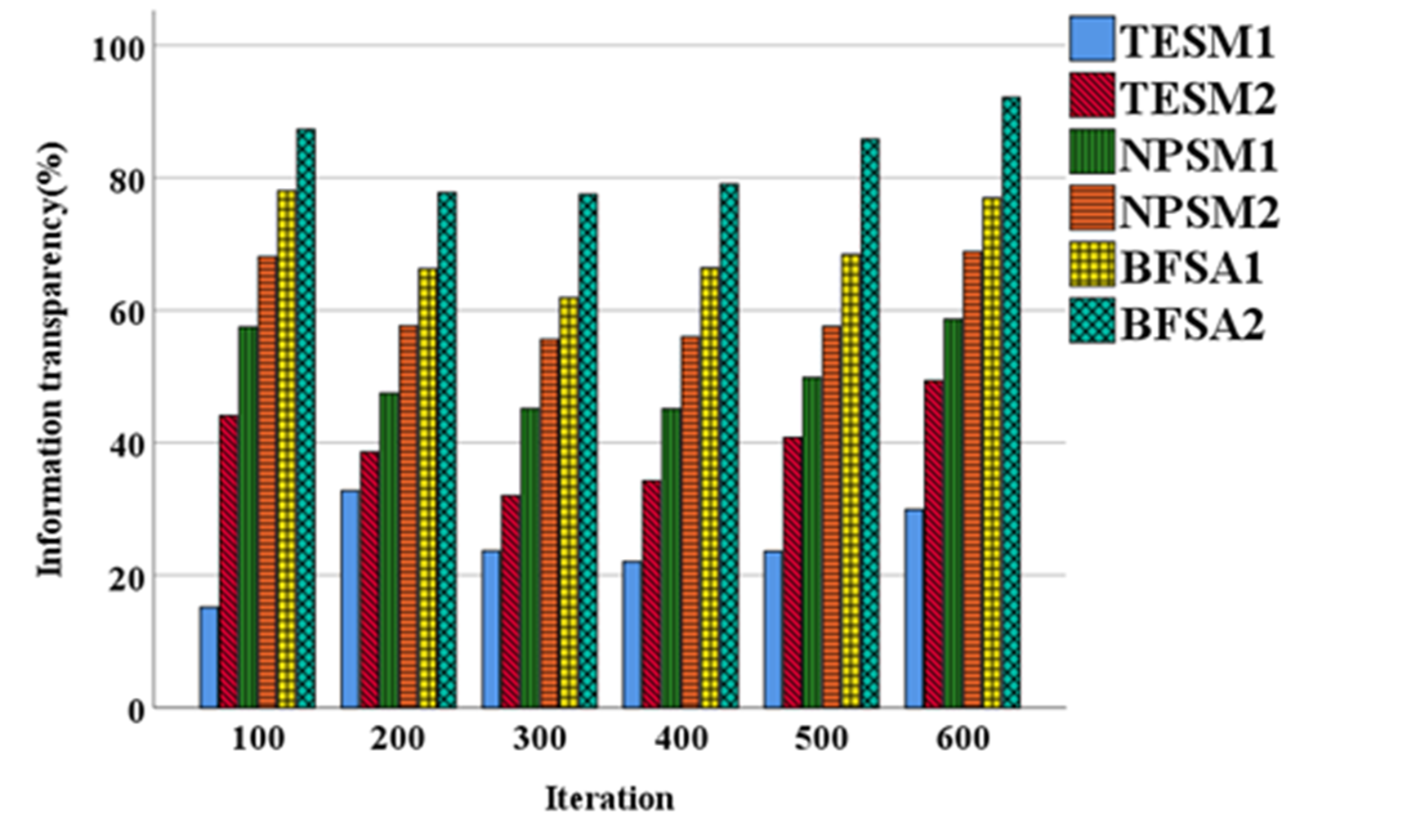

Supplement: S1 Data — (ZIP) [file pone.0298210.s001.zip › ╩2╛▌░n/Figures/figure8.tif]
